# Opportunities and Challenges of Tobacco Control Policy at District Level in Indonesia: A Qualitative Analysis

**DOI:** 10.31557/APJCP.2021.22.10.3055

**Published:** 2021-10

**Authors:** Sepri Yunarman, Aries Munandar, Abdillah Ahsan, Ali Akbarjono, Dian Kusuma

**Affiliations:** 1 *Faculty of Tarbiyah and Tadris, Institut Agama Islam Negeri Bengkulu, Bengkulu, Indonesia. *; 2 *Faculty of Social and Political Science, University of Bengkulu, Bengkulu, Indonesia. *; 3 *Faculty of Economics and Business, University of Indonesia, Depok, Indonesia. *; 4 *Centre for Health Economics & Policy Innovation, Imperial College Business School, London, UK. *

**Keywords:** Tobacco control, smoke-free policy, opportunity, challenge, Indonesia, Bengkulu

## Abstract

**Introduction:**

Comprehensive tobacco control policies are lacking in Indonesia where smoking prevalence in males is among the highest in the world. This study aims to explore the knowledge, attitude, opportunities and challenges to tobacco control among local stakeholders.

**Methods:**

This is a qualitative study using in-depth interviews. Four study areas included Bengkulu Province, Bengkulu City, Seluma District, and Kaur District. Eighteen participants interviewed were from policymakers, legislators, and civil societies during November-December 2020. Thematic data analysis was used.

**Results:**

While knowledge and support of the existing Smoke Free Policy (SFP) were high, that of other policies such as outdoor tobacco advertising (OTA) ban and tobacco product display ban were low. Among others, one opportunity was there is already SFP regulation in each study area, to which such bans can be added. Among others, three major challenges were: (a) lack of enforcement of the existing SFP, (b) lack of national regulation to ban OTA and product display, and (c) counter actions by the tobacco industry.

**Conclusion:**

The opportunities and challenges identified could be lessons learnt for more comprehensive tobacco control especially by local governments in Indonesia and other countries with similar settings.

## Introduction

Indonesia, a country in the Southeast Asia region with population of over 270 million, is one among only nine countries in the world that are neither signatories nor parties to the Framework Convention on Tobacco Control (FCTC) (World Health Organization [WHO], 2020). In effect, comprehensive tobacco control policies are lacking in the country where smoking prevalence among men (15+ years) is among the highest in the world at 67% and that among boys (13-14 years) is high at 36.2% (WHO, 2018; Ministry of Health [MOH], 2011). 

At the national level, the only major tobacco control policy is the smoke-free policy (SFP), which is based on the Health Act 36/2009 and Presidential Decree 109/2012 (President of Indonesia, 2012). The former is a recommendation for local governments to adopt SFP and the latter provides the details. The law prohibits production, sales, advertisement, promotion, and active smoking of tobacco products at certain facilities such as offices and schools. Data show slow policy adoption with only two-thirds of districts (345 of 514) enacted a form of SFP by December 2018, with low compliance rate in many areas including Jayapura city (17% compliance rate) (Wahidin et al., 2020; Wahyuti et al., 2019).

However, national regulations to ban outdoor tobacco advertisement (OTA) and tobacco product display at the point-of-sale (POS) are nonexistent. Consequently, only a handful districts in the country (approximately 10% of districts by 2018) have piloted or implemented the bans, including Bogor City (Priyono et al., 2020). The city government initially focused on effective implementation of SFP and reached high compliance by 2014 (No Tobacco Community, 2014). Then, it banned new permits of OTA during 2014-2015 and enacted the ban of tobacco displays at POS in 2017. The ban, which started among modern chain retailers, included covering tobacco products and removing posters at POS (Priyono et al., 2020). Similarly, the neighboring Depok City implemented OTA ban in 2018 and piloted display ban in 2019 (Government of Depok City, 2020). 

Previous studies have attempted to explore factors that contribute to the slow progress of tobacco policy change in Indonesia from the perspective of national tobacco control experts (Astuti et al., 2020). However, such evidence is lacking at the local government level (i.e. provincial and district level). Thus, our study aims to explore the knowledge, attitude, opportunities and challenges to more comprehensive tobacco control efforts in the country, taking Bengkulu Province as an example (along with three of its city/district governments). Bengkulu is among the poorest provinces and the highest smoking prevalence in the country. The Basic Health Survey (Riskesdas) 2018 data show 31.9% of population aged 10+ years smoked, just below West Java Province (32.3%) and Gorontalo Province (32.0%).

## Materials and Methods

**Figure 1:**
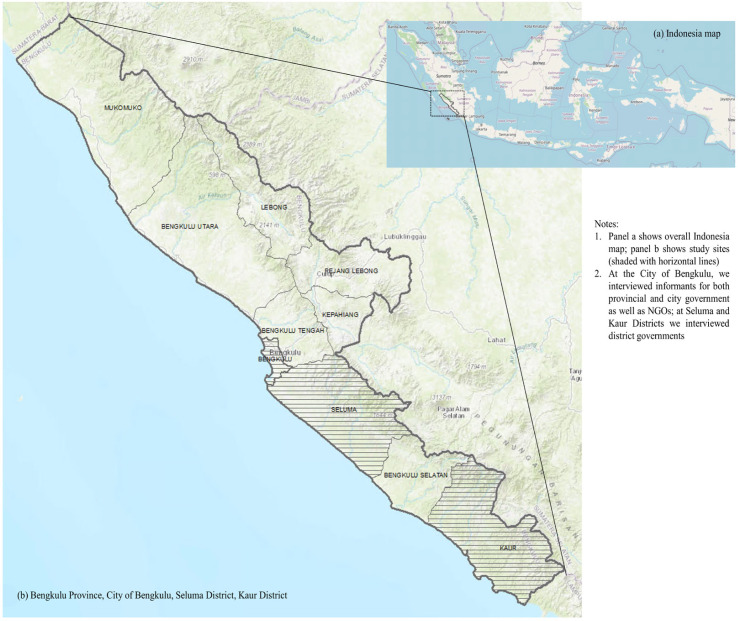
Study Site

This is a qualitative study using in-depth interviews. There were four study settings including Bengkulu Province, Bengkulu City, Seluma District, and Kaur District. Within the province, there are ten city/districts which all have adopted the SFP policy through a regulation by local legislator (DPRD). Study settings were purposively selected: Bengkulu City is the provincial capital; Seluma District is rural (i.e. district) but adjacent to the capital; and Kaur district is far from the capital bordering with other provinces (Figure 1). 

Participants were key stakeholders relevant to local tobacco control efforts. They included policymakers (Provincial/District Health Office, Civil Servant Policy (Pol PP), and Local Planning Agency (Bappeda); legislators (DPRD), and non-government organizations (NGOs). Participants were purposively selected to ensure representation of different key stakeholders relevant to tobacco control efforts at the provincial and district level. Eighteen eligible participants were identified and were contacted by the interviewer. 

Data collection was performed by the first author, using a semi structure interview guide developed by the research team. The aim and procedure of the study were explained before asking participants for their willingness and consent to participate in the interviews. Verbal informed consent was obtained from all participants prior to the interviews. Because of COVID-19 restriction, the interviews were conducted by phone. The duration of interviews was approximately 20 minutes. Interviews were conducted in mixed Indonesian and Bengkulu language by the interviewer. The first author is fluent in both languages. All interviews were digitally recorded. 

The digital recordings of all interviews were transcribed verbatim into Indonesian by the interviewer. Thematic analysis was performed on the responses. The first and last authors read the transcripts line-by-line independently and assigned codes to meaningful responses. Codes were developed and recurrent codes were grouped into themes based on discussions among all authors and relevant literature. Themes were categorized into reasons for support, opportunity, and challenges of more comprehensive tobacco control at the provincial and district level. Data analysis was conducted using Microsoft Word and Excel.

Ethical clearance was obtained from the Ethics Committee for Health Research, University of Hasanuddin Faculty of Public Health, Makassar, Indonesia (Number 7138/UN4.14.1/TP.02.02/2020). 

## Results

Table 1 shows the characteristics of participants. Of the 18 participants interviewed, 17 were males and 1 were females. By institution, four participants were from Provincial/District Health Office, four from Civil Servant Police, four from Planning Agency, four from Parliament, and two from NGOs. By smoking status, 12 participants do not smoke and six participants currently smoke. 

In terms of knowledge, there are two types of tobacco control policies discussed during the interviews. First, the current SFP policy in each study setting (province, city, and district). Second, in addition to SFP, more comprehensive policies to ban outdoor tobacco advertising (OTA) and tobacco display at point of sales in Bogor City and Depok City (West Java Province). All 18 participants reported knowing the current SFP regulation in Bengkulu Province and/or each city/district. However, only five participants knew or ever heard of OTA ban and display ban in Bogor City and Depok City. Furthermore, 10 participants expressed support of the local SFP regulation. Five participants expressed full support on the OTA ban and display ban while five participants reported ‘partial’ support, with conditions or considerations.

*Reasons for support (or lack thereof) to tobacco control policies*

Table 2 shows the summary of thematic analysis in terms of support (panel a), lack of support (b), opportunities (c), and challenges (d) to more comprehensive tobacco control policies such as SFP, OTA ban, and display ban. From the interviews, there are at least six reasons for support to the current SFP regulation and/or more comprehensive tobacco control policies including OTA ban and display ban. They include: (1) Health reason, personal experience better health after stopped smoking; (2) To avoid risk of secondhand smoking (SHS) for non-smokers (home, school, office); (3) To prevent young people smoking (home, schools); (4) Policy/regulation needed because behavioral change is difficult and takes time; (5) Policy/regulation needed to raise awareness in community; and (6) Because those are government policy (e.g. OTA ban and display ban in Bogor, Depok). 

“*We need discipline in smoking, to avoid risks for others (non-smokers)*.” (P11) 

“*I support more comprehensive policies because behavior change (smoking) is really difficult. We need to keep raising community awareness.*” (P15)

While all participants expressed support to the current SFP regulation, many expressed lack of full support to the OTA ban and display ban implemented in Bogor City and Depok City. They include: (1) Need to consider economic, social implications; (2) potential impact on farmers; (3) the policies are difficult to implement; (4) the province, city, and districts need the additional income from tobacco sale and advert tax; (5) the lack of national policy to ban OTA and display.

“*Indeed, in the healthy city competition, one of the points is to reduce the number of outdoor tobacco advertising. However, we need the income from the advert tax. Also, we should not forget that we get billion rupiah from tobacco sales tax. So, it’s a dilemma.*” (P9)

*Opportunities and challenges for more comprehensive tobacco control*

Participants reported at least four opportunities for more comprehensive tobacco control in the province and districts. They include: (1) Mayor does not smoke, potential support; (2) SFP regulation is a national program; (3) There are SFP regulation in place in province and districts, only needs to revise content; and (4) Potential for area-limited OTA ban and display ban such as around schools.

“*(Regarding OTA ban and display ban) I think we do not need another legislation, use the current SFP regulation and add the content. We also need to make sure the current SFP is implemented effectively.*” (P2)

However, there are many challenges towards more comprehensive tobacco control in the province and district. They include: (1) Smoking is cultural in the community for a long time; (2) So many smokers in the community, policymakers, legislators; (3) National regulation to ban OTA and display is needed; (4) Lack of understanding on danger of smoking in community; (5) SFP implementation lack of enforcement: mostly limited to monitoring but no penalty, lack funding; (6) SFP lack of funding to enforce, lack of resource to create smoke-only room in offices; (7) SFP community hesitant to heckle violators (e.g. smoking indoor); (8) Needs studies involving multisectoral: provincial gov, district gov, NGO, traders, experts; (9) Needs income from tobacco tax and OTA (high income areas may be able to afford OTA ban); and (10) Opposition from tobacco industry.

“*We cannot do it (at the local level) without policy/regulation at the higher level (e.g. national, provincial). Personally, I support the policy (OTA ban, display ban) but we need the regulation at higher level, currently does not exist.*” (P14)

**Table 1:** Characteristics of Participants

Participant	Gender	Study setting	Institution
1	Male	Bengkulu Province	Health Office
2	Male	Bengkulu Province	Civil service police
3	Male	Bengkulu Province	Planning Agency
4	Male	Bengkulu Province	Parliament
5	Male	Bengkulu Province	NGO
6	Male	Bengkulu Province	NGO
7	Male	Bengkulu City	Health Office
8	Male	Bengkulu City	Civil service police
9	Male	Bengkulu City	Planning Agency
10	Male	Bengkulu City	Parliament
11	Male	Seluma District	Health Office
12	Male	Seluma District	Civil service police
13	Male	Seluma District	Planning Agency
14	Male	Seluma District	Parliament
15	Male	Kaur District	Health Office
16	Male	Kaur District	Civil service police
17	Male	Kaur District	Planning Agency
18	Female	Kaur District	Parliament

Note: Health Office is Provincial Health Office or District Health Office; Civil Service Police is Polisi Pamong Praja (Pol PP); Planning Agency is Bappeda; Parliament is DPRD; and NGO is non-government organization. The positions of participants in their institution varied from echelon 3, echelon 2, and head

**Table 2:** Summary of Reasons, Opportunities, and Challenges for Comprehensive Tobacco Control

(a)	Why support more comprehensive tobacco control
1	Health reason, personal experience better health after stopped smoking
2	To prevent risk of secondhand smoking (SHS) for non-smokers (home, school, office)
3	To prevent young people from smoking (home, schools)
4	Policy/regulation needed because behavioral change is difficult and takes time
5	Policy/regulation needed to raise awareness in community
6	Because those are government policy (e.g. OTA ban and display ban in Bogor, Depok)
(b)	Why partial support or lack of support for more comprehensive tobacco control
1	Need to consider economic, social implications
2	Potential impact on farmers
3	The policies are difficult to implement
4	The province, city, and districts need the additional income from tobacco sale and advert tax
5	The lack of national policy to ban OTA and display.
(c)	Opportunity for more comprehensive tobacco control at province/district
1	Mayor does not smoke, potential support
2	SFP regulation is a national program
3	There are Smoke Free Policy regulation in place in province and districts, only needs to revise content
4	Potential for area-limited OTA ban and display ban such as around schools
(d)	Challenges for more comprehensive tobacco control at province/district
1	Smoking is cultural in the community for a long time
2	So many smokers in the community, policymakers, legislators
3	National regulation to ban OTA and display is needed
4	Lack of understanding on danger of smoking in community
5	SFP implementation lack of enforcement: mostly limited to monitoring but no penalty, lack funding
6	SFP lack of funding to enforce, lack of resource to create smoke-only room in offices
7	SFP community hesitant to remind violators (e.g. smoking indoor)
8	Needs studies involving multisectoral: provincial gov, district gov, NGO, traders, experts
9	Needs income from tobacco tax and OTA (high income areas may be able to afford OTA ban)
10	Opposition from tobacco industry

SFP, Smoke Free Policy (Kawasan Tanpa Rokok); OTA, Outdoor tobacco advertising; SHS, Secondhand Smoking; NGO, Non-Government Organization

## Discussion

We found that knowledge and support on the existing SFP regulation was high among participants who are policymakers, legislators, and civil society. This is partly because SFP that has been enacted since 2012 nationally is the only major national regulation on tobacco control in the country (President of Indonesia, 2012). However, because the responsibility to implement is given to provincial and district governments, policy adoption has been slow (Wahidin et al., 2020). In fact, our study participants have identified the lack of enforcement and of funding of SFP policy were observed at the provincial and district level. Currently, implementation was limited to monitoring compliance but no (financial) penalty in place in Bengkulu. Although some participants noted experience of a more effective enforcement in Jakarta. The lack of funding reduced the ability to enforce compliance and to create smoke-only room in offices. Also, the community was hesitant to remind violators of SFP such as smoking indoor (Kaufman et al., 2015).

In contrast, knowledge and support among participants on OTA ban and display ban, as two examples of more comprehensive tobacco control, relatively low. This is partly because there is currently no national policy on OTA ban and display ban. In effect, only a handful of districts in the country implementing the bans, including Bogor City and Depok City. Participants expressed dilemma to support such bans. On one hand, many understand the danger of smoking to smokers and non-smokers. On the other hands, participants asserted the need for additional government income from tobacco tax and outdoor advertising tax. A participant also noted that high income districts may be able to afford OTA ban, because of smaller reliance to such additional income (Sebayang et al., 2018). 

 Our study elicits fewer opportunities than challenges to more comprehensive tobacco control policies. One potential opportunity is that in all study settings, there are already SFP regulation in place. Given the difficulty political process in making a legislation including at the provincial and district level, some participants suggested to revise the current SFP regulation to include more comprehensive tobacco control policies such as OTA ban and display bans (instead of creating a new regulation for each ban). In fact, this was exactly what the government of Bogor City did (i.e. adding the OTA ban and display ban into the existing SFP regulation) (Priyono et al., 2020). Another potential opportunity is to start the bans in more limited areas such as around schools. This may be appealing to most members of the community and policymakers to protect young people to become smokers.

In terms of challenges, two major challenges expressed by study participants were that smoking is cultural in the community and so many smokers among the community, policymakers, and legislators. Participants mentioned the need to avoid protest from the community if such bans are seen to tough. Regulations for such bans maybe difficult also because many legislators are smokers themselves. Another major challenge is the lack of national regulation to ban OTA and display by the central government (Kusuma et al., 2019). While local government may take the initiative (e.g. Bogor City and Depok City), many legislators and policy makers especially those without health background may not be easily convinced to taking such initiative, without the existence of higher-level regulation (or mandate). Another major challenge would be the potential opposition from the tobacco industry (Assunta and Dorotheo, 2016). When the government of Bogor City implemented the OTA ban and display ban, tobacco industry representatives challenged in the supreme court (Supriyanto et al., 2020). This may serve as a warning to other district governments that may be considering adopting the bans.

This evidence from our study is relevant for policymakers and stakeholders at the national, provincial, and district level in Indonesia towards more comprehensive and effective tobacco control efforts. The opportunities and challenges identified should be lessons learnt for better implementation of the existing SFP policy and potential adoption of other policies such as OTA ban and display ban at the local government level. Our study, however, has limitations in terms of interview process (by phone during COVID-19 pandemic) and relatively limited study setting. Further study may employ video call interview to improve interaction with participants may be conducted in more provinces and districts (among 500+ district governments) in the country. Despite the limitations, however, findings of our study are relevant for Indonesia and other lower income countries aspiring towards more comprehensive and effective tobacco control.

## Author Contribution Statement

SY, AM, AA and DK conceptualized the study. SY, conducted data collection. SY and DK analyzed the data. DK drafted and SY, AM, and AA provided inputs to the manuscript. All authors approved the final version of the manuscript.
